# TNF and regulatory T cells are critical for sepsis‐induced suppression of T cells

**DOI:** 10.1002/iid3.75

**Published:** 2015-08-04

**Authors:** David Stieglitz, Tobias Schmid, Nirav F. Chhabra, Bernd Echtenacher, Daniela N. Männel, Sven Mostböck

**Affiliations:** ^1^ Institute of ImmunologyUniversity of RegensburgRegensburgGermany

**Keywords:** Immunosuppression, suppressor cells, TNF receptors, Treg

## Abstract

The immune system in sepsis is impaired as seen by reduced numbers and function of immune cells and impaired antigen‐specific antibody responses. We studied T cell function in septic mice using cecal ligation and puncture (CLP) as a clinically relevant mouse model for sepsis. The proliferative response of CD4^+^ and CD8^+^ T cells was suppressed in septic mice. Adoptive transfer experiments demonstrated that the T cells were not intrinsically altered by CLP. Instead, the septic host environment was responsible for this T cell suppression. While CLP‐induced suppression was dependent on TNF activity, neither the activation of TNF receptors type 1 nor TNF receptor type 2 alone was sufficient to generate sepsis‐induced suppression showing that the two TNF receptors can substitute each other. Specific depletion of regulatory T (Treg) cells improved the impaired T cell proliferation in septic recipients demonstrating participation of Treg in sepsis‐induced suppression. In summary, sepsis leads to TNF‐dependent suppression of T cell proliferation in vivo involving induction of Treg cells.

## Introduction

In sepsis the host immune system responds with a complex interplay of pro‐ and anti‐inflammatory processes 1. For a long time it was the prevailing opinion that an initial inflammatory immune response is followed by a compensatory anti‐inflammatory response to reconstitute immune homeostasis 2. Others and more recent results, however, demonstrate that early in sepsis both types of immune reactions occur simultaneously 3, 4, 5. Both immune reactions contribute to clearance of infection and tissue recovery but also bear the risk of organ injury and secondary infections 6. A suppressed immune status in sepsis is characterized by depletion of immune cells, development of suppressive myeloid cells (MDSC), and increased numbers of regulatory T cells (Treg). In patients who died of sepsis marked signs of immune suppression were observed such as decreased cytokine production and expansion of Treg and MDSC 7. As efficient adaptive immune responses are prerequisites to control infection, sepsis‐induced immune deviation comprises the danger of opportunistic infections and reactivation of latent virus 8. Patients recovering from sepsis remain at risk for a prolonged time and, therefore, sepsis‐induced immunosuppression represents a clinical problem 9.

The model of cecal ligation and puncture (CLP) is a clinical relevant mouse model for poly‐microbial septic peritonitis 10. In this experimental model for sepsis, we were previously able to demonstrate the above described sepsis‐derived immune deviations such as reduced cytokine production 11, 12, 13, impaired functionality of dendritic cells (DC) 14, and the increased ratio of Treg in the CD4^+^ T cell population 15. Further, we showed that the primary B cell response in septic mice was impaired 16. Additionally, the induction of MDSC in sepsis was demonstrated in the CLP model by others 17.

The efficiency of an adaptive immune response critically depends on the effector functions of T cells. As our previous work established the impact of sepsis on antigen‐presenting cells and B cells, we aimed this study on completing the view on sepsis by testing T cell function directly in vivo. Here, we demonstrate that T cell proliferation is impaired following sepsis. This effect is neither based on intrinsic changes in the T cells, nor on reduced function of antigen‐presenting cells. Instead, the inflammatory cytokine TNF and Treg cells were shown to cause reduced T cell function.

## Results

### Sepsis induces suppression of in vivo T cell proliferation

To analyze the systemic impact of sepsis on T cell function in CLP‐treated mice, we focused on their proliferative capacity as a surrogate marker for T cell effector function. In general, we purified splenic T cells, labeled them ex vivo with CFSE, a marker for proliferation, and transferred them into host mice, either naïve mice or mice that had been subjected to CLP the day before.

**Figure 1:**
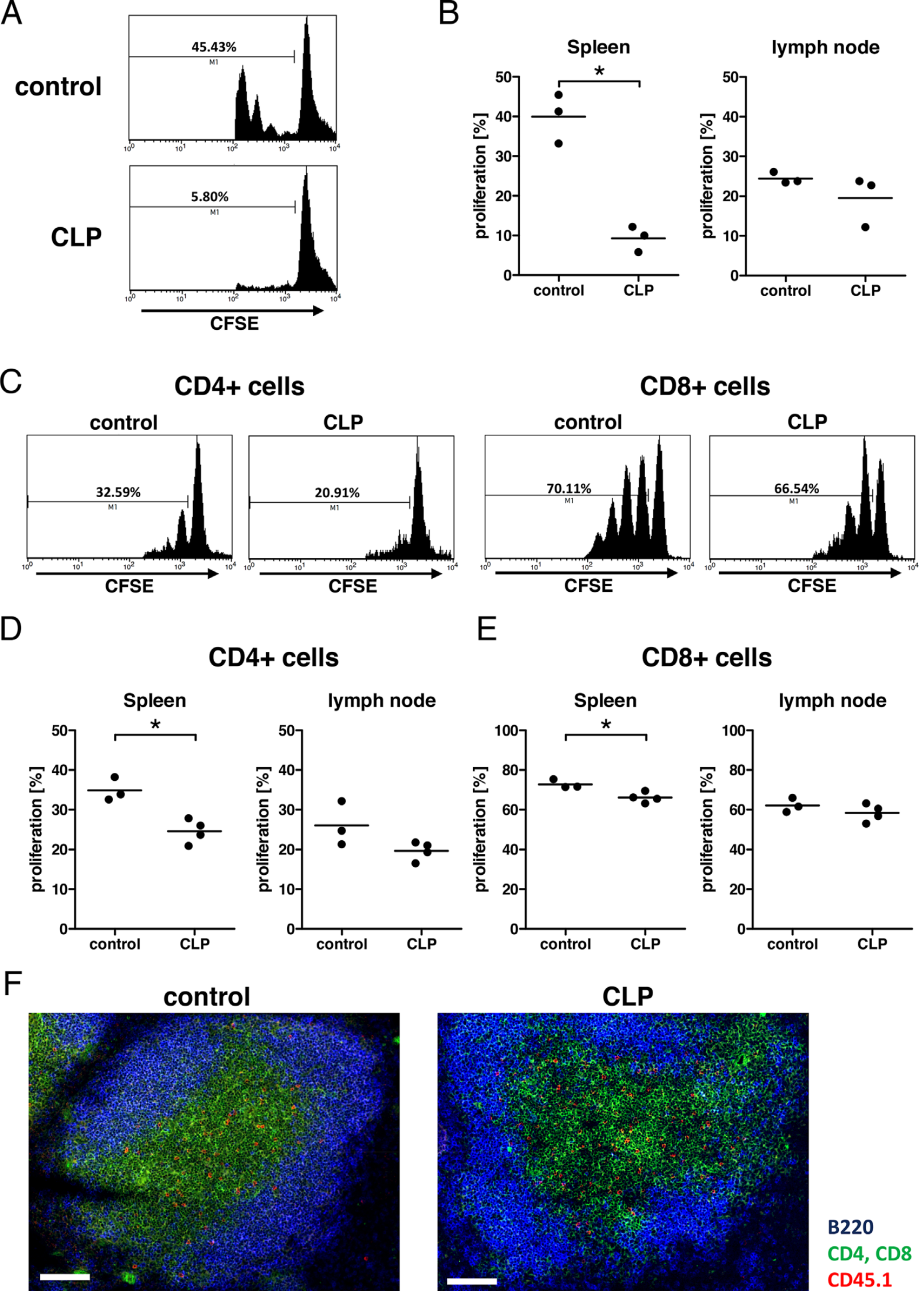
T cells in CLP‐exposed mice demonstrate reduced proliferation in vivo. (A+B) CD4^+^ T cells were prepared from OT‐II mice, labeled with CFSE, and transferred into untreated control C57BL/6 mice or C57BL/6 mice 2 days after CLP. The day after transfer, Ovalbumin was injected s.c. into the abdomen of the mice. Three days later, the draining inguinal lymph nodes and the spleens were harvested and CFSE‐positive CD4^+^ T cells analyzed by flow cytometry. (A) CFSE histogram of a representative spleen of a control mouse (upper panel) and a CLP‐treated mouse (lower panel). M1 indicates the marker identifying proliferating cells. (B) Percentages of proliferating cells of the CFSE‐positive population in spleen and lymph nodes of control and CLP‐treated mice. Each symbol represents one mouse, horizontal bar is the mean. * indicates *P* < 0.05 in Student's *t*‐test. This experiment was performed once. (C–E) T cells were prepared from untreated C57BL/6 mice, labeled with CFSE, and transferred into untreated control C57BL/6 mice or C57BL/6 mice 2 days after CLP. The day after transfer, anti‐CD3ϵ antibody was injected i.v. into the mice. Three days later, the inguinal lymph nodes and the spleens were harvested and CFSE‐positive T cells analyzed by flow cytometry. (C) CFSE histogram of CD4^+^ T cells (left two panels) and CD8^+^ T cells (right two panels) from a representative spleen of a control mouse (respective left panel) and a CLP‐treated mouse (respective right panel). M1 indicates the marker identifying proliferating cells. (D,E) Percentages of proliferating CFSE‐positive (D) CD4^+^ cells and (E) CD8^+^ cells in spleen and lymph nodes of control and CLP‐treated mice. Data shown are from one representative experiment; each symbol represents one mouse, horizontal bar is the mean. * indicates *P* < 0.05 in Student's *t*‐test. This experiment was performed five times with similar results, twice with i.v. injection of the antibody and three times with i.p. injection of the antibody. (F) T cells were prepared from untreated CD45.1 mice and transferred into untreated control C57BL/6 mice (left panel) or C57BL/6 mice 2 days after CLP (right panel). The day after transfer, spleens were harvested and analyzed in fluorescence immunohistology (blue: B220^+^ cells, green: CD4^+^ and CD8^+^ cells, red: CD45.1 cells; magnification 200×, white bar = 100 μm). This experiment was done once.

In order to determine the antigen‐specific proliferative capacity of CD4^+^ T cells in vivo, mice received CFSE‐labeled CD4^+^ T cells with a T cell receptor (TCR) specific for ovalbumin (OT‐II CD4^+^ T cells). One day after T cells transfer, the mice were immunized with ovalbumin and the T cell proliferation was determined in the splenic and lymph node CD4^+^ T cell population 3 days later (Fig. 1A). Proliferation of the transferred CD4^+^ T cells was strongly reduced in the spleen of septic recipient mice compared to naïve recipient mice (Fig. 1B).

Considering our previous finding of impaired DC function in septic mice, 14 we decided to activate wild‐type T cells in a DC‐independent fashion by using anti‐CD3ϵ antibody treatment in the following experiments. This treatment activates CD3ϵ, a part of the TCR complex, which then starts the TCR signaling pathway. CD3ϵ activation is one of the first events in the natural activation of T cells. Hence, the activation of T cells by anti‐ CD3ϵ antibodies follows the natural activation of T cells closely. Very similar to the results with antigen‐specific stimulation, the in vivo proliferation of adoptively transferred T cells was reduced upon non‐specific T cell activation with anti‐CD3 in septic mice (Fig. 1C). CLP exerted a more pronounced suppressive effect on the proliferative response of CD4^+^ T cells compared to CD8^+^ T cells (Fig. 1D, E). In addition, the sepsis‐induced repression of the proliferative T cell response was more clearly visible with T cells derived from spleens compared to peripheral lymph nodes. Therefore, only the results with splenic T cells will be shown in further experiments.

We wondered whether the reduced proliferative response of T cells in septic mice might be based on an altered splenic architecture following sepsis. However, while the spleen size was reduced in septic mice as we have shown previously 16, we did not observe a difference in morphology of the spleen nor in the location of T cells in the spleen between naïve and septic mice after T cell transfer (Fig. 1F).

**Figure 2:**
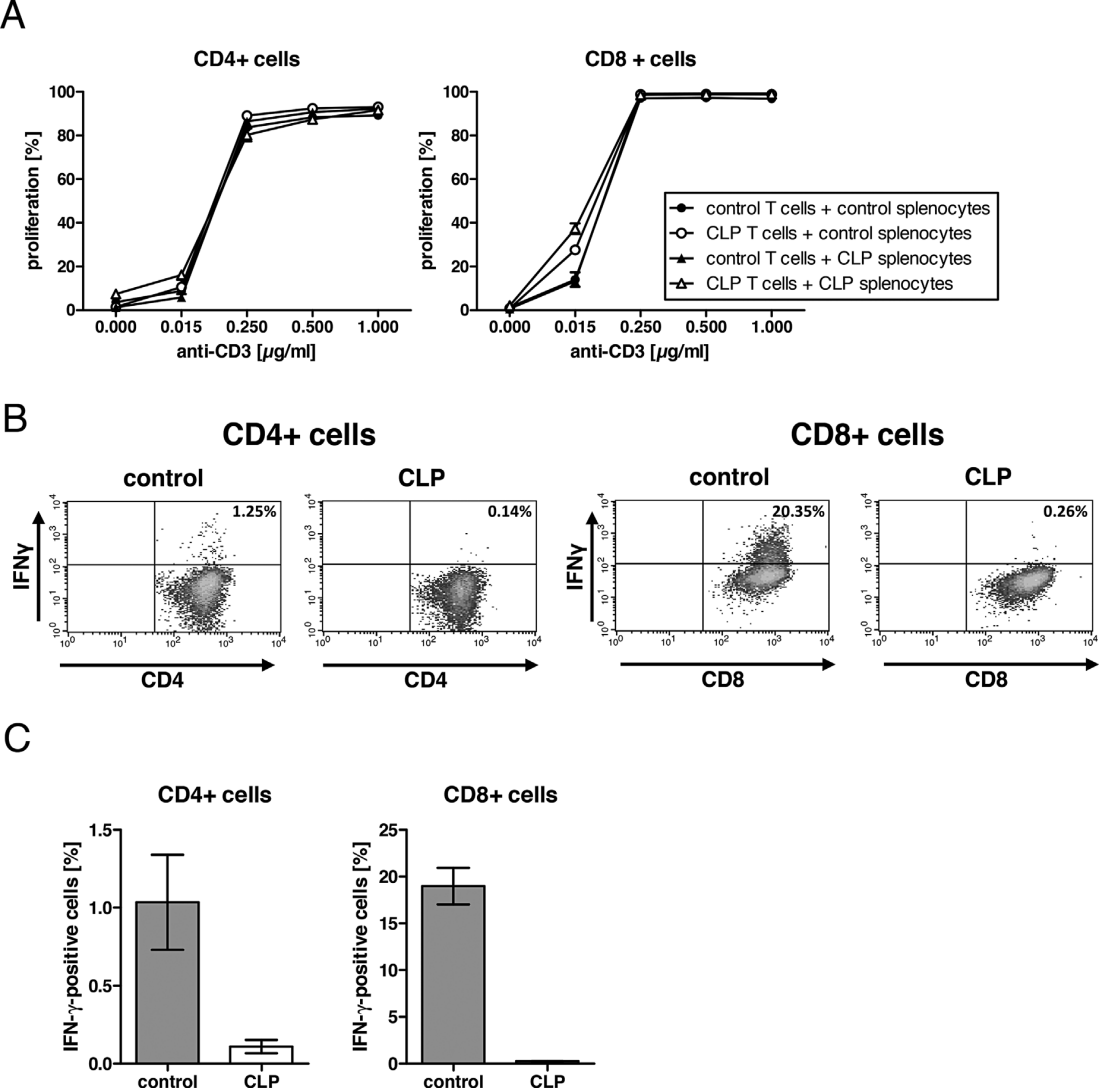
T cells from CLP‐exposed mice demonstrate reduced cytokine production in vitro. (A) T cells were separated from splenocytes of CD45.1 mice or from C57BL/6 mice two days after CLP, labeled with CFSE and cultured in presence of either splenocytes from control untreated CD45.1 mice or from C57BL/6 mice two days after CLP. After three days in culture with varying concentrations of soluble anti‐CD3ϵ antibody, cells were analyzed by flow cytometry to determine proliferating cells. Data are from a representative experiment and shown as mean +/− standard deviation of three culture well replicates. The experiment was done twice. (B‐C) Total splenocytes from either naïve control C67BL/6 mice or C57BL/6 mice two days after CLP were cultured for 24 h with 0.5 μg/ml soluble anti‐CD3ϵ antibody and analyzed for IFN‐γ‐producing cells by flow cytometry. (B) Representative density plots and (C) data of two culture well replicates (mean +/− SD) are shown. The experiment was done once.

As T cell proliferation was reduced in vivo even after antigen‐unspecific activation, we were interested in the impact of other cells of the immune system on T cell effector function. Labeled splenic T cells from either naïve or septic mice 2 days after CLP were tested for their proliferative capacity ex vivo in the presence of unlabeled splenocytes from either naïve or septic mice upon stimulation with anti‐CD3 antibodies (Fig. 2A). To mimic the in vivo application of anti‐CD3 antibody, we added the antibody soluble to the culture. The in vitro proliferation of CD4^+^ and CD8^+^ T cells was very similar in all experimental settings and no sepsis‐dependent impairment was observed. Only at suboptimal concentrations of anti‐CD3 antibodies T cells from septic mice seemed to respond slightly more sensitive to the stimulation compared to the T cells from naïve mice. The contact with splenocytes from either naïve or septic mice did not affect the T cell proliferation differentially. The data clearly show that T cell activation and proliferation in vivo in septic mice was impaired while the proliferative response of T cells ex vivo from septic mice was not different compared to the proliferation of T cells from naïve mice.

However, the production of IFNγ by CD4^+^ and CD8^+^ T cells in whole splenocyte preparations from septic mice 2 days after CLP was strongly reduced upon in vitro stimulation with anti‐CD3 antibodies (Fig. 2B and C), reflecting the previously observed impaired cytokine production in sepsis 12.

### Sepsis confers suppressive host effect on transferred T cells

**Figure 3:**
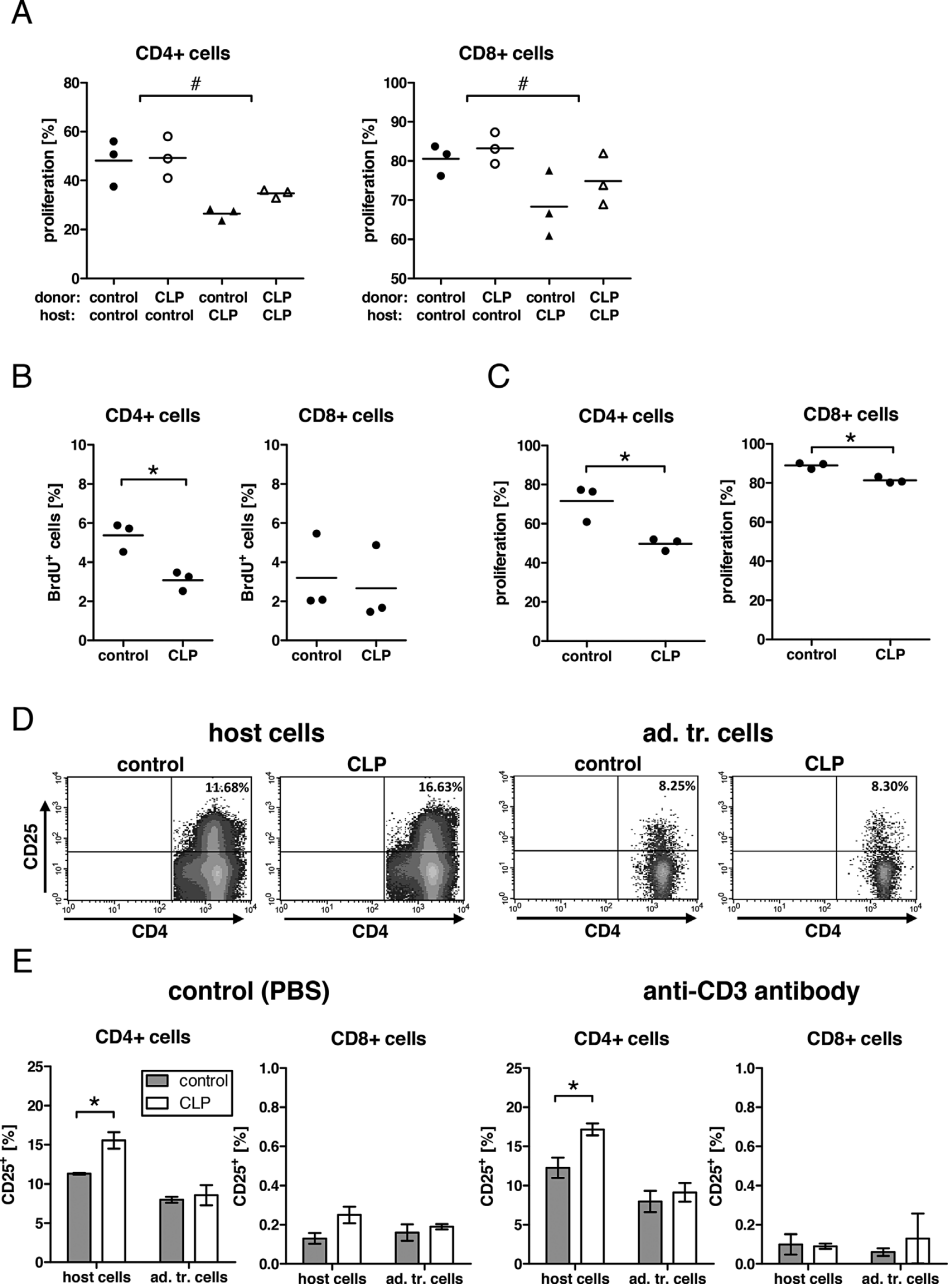
The detrimental effect on T cell‐function in vivo depends on the host environment. (A and B) Splenocytes were prepared from untreated control C67BL/6 mice or C57BL/6 mice 2 days after CLP. T cells were separated from the splenocytes, labeled with CFSE, and injected into either control untreated C57BL/6 mice or from C57BL/6 mice 2 days after CLP. The next day, animals received an i.p. injection of anti‐CD3ϵ‐antibody and BrdU. Three days later, spleens were harvested and analyzed by flow cytometry for proliferating cells based on (A) CFSE dilution of the CFSE‐positive population or (B) BrdU incorporation of host cells. Each symbol represents one mouse, line shows the mean. # indicates that CLP in the host, but not in the donor animals, was a statistically significant factor in a two‐way ANOVA. * indicates *P* < 0.05 in Student's *t*‐test. Experiment was performed once. (C) Splenocytes were prepared from untreated control C57BL/6 mice. T cells were separated from the splenocytes, labeled with CFSE, and injected into untreated C57BL/6 mice. One day after the adoptive transfer, one group of animals was exposed to CLP. Four days after the adoptive transfer, all mice received an i.p. injection of anti‐CD3ϵ‐antibody. Two days after injection, spleens were harvested and analyzed by flow cytometry for proliferating cells of the CFSE‐positive population. Each symbol represents one mouse, line shows the mean. * indicates *P* < 0.05 in Student's *t*‐test. Experiment was performed once. (D and E) Splenocytes were prepared from untreated control C57BL/6 mice. T cells were separated from the splenocytes, labeled with CFSE, and injected into untreated C57BL/6 mice or C57BL/6 mice 2 days after CLP. The next day, mice received either PBS as a control (E—left two panels) or anti‐CD3ϵ‐antibody (D and E—right two panels) in an i.p. injection. Three days after injection, splenocytes were harvested and analyzed by flow cytometry for the level of CD25‐positive cells in host and adoptively transferred cells (identified as CFSE‐positive cells). (D) Density plots of the CD4^+^ cells of one representative mouse per group (anti‐CD3ϵ‐antibody treatment) and (E) data from 2–3 mice per group (mean +/− SD) are shown. * indicates *P* < 0.05 in Student's *t*‐test. One representative experiment of two is shown.

Transfer experiments were performed in order to test whether the suppressive effect on T cell proliferation in septic mice relied on the T cells themselves or on the host environment. Either labeled naïve T cells or labeled T cells from septic mice were transferred into either naïve or septic mice. Very clearly, only the transfer into septic hosts conferred suppression while the origin of the transferred T cells did not have an impact (Fig. 3A). This finding established the septic host environment to exert the suppressive effect on T cell proliferation. In addition, the proliferative capacity of the host cells during the time of the transfer experiment (from day 2 until day 6 post CLP) was also impaired as measured by reduced BrdU uptake (Fig. 3B). Transfer of labeled T cells 1 day prior to CLP also resulted in a sepsis‐dependent suppression of proliferation (Fig. 3C), indicating that exposure to the entire time span from the time point of CLP induction (from day 0 to day 5 post CLP) did not change the suppressive effect on T cell proliferation as long as the activating stimulus was given after CLP. Together these results show that the environment in the septic host is responsible for the impaired T cell proliferative response.

However, we observed a differential effect of CLP on the expression of the high‐affinity IL‐2 receptor chain CD25 on host‐derived and transferred T cell populations. Six days after CLP, only recipient CD4^+^ cells contained a statistically significant increased proportion of CD25^+^ cells, while CD25 expression on the transferred CD4^+^ was not enhanced. No significant changes were observed in the CD8^+^cells (Fig. 3D and E).

### Activation of both TNF receptors is required for sepsis‐induced suppression

The TNF system has been demonstrated previously to play an important role during sepsis by being involved in Treg generation or function 15, 18. T cells genetically deficient for TNF production were used to determine the impact of TNF on the reduced T cell proliferation following sepsis.

**Figure 4:**
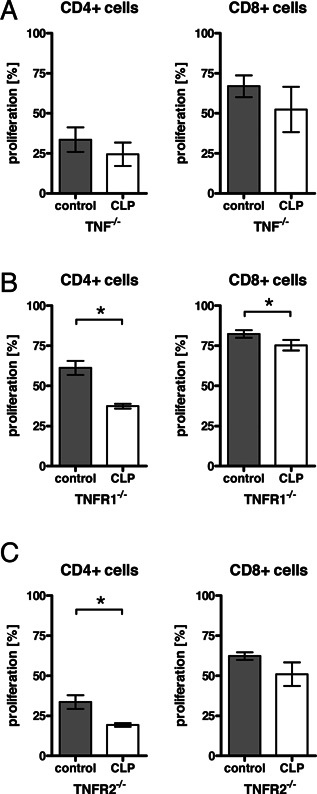
TNF is not involved in the reduced proliferative capacity of T cells in CLP‐exposed animals. Splenocytes were prepared from untreated control mice. T cells were separated from the splenocytes, labeled with CFSE, and injected into untreated mice or mice 2 days after CLP. The next day, mice received anti‐CD3ϵ‐antibody. Three days after injection, splenocytes were harvested and analyzed by flow cytometry for proliferating cells of the CFSE‐positive population. (A) T cells were prepared from TNF‐deficient mice and transferred into TNF‐deficient mice. Anti‐CD3ϵ‐antibody was given in an i.v. injection. Data from 2–3 mice per group are shown as mean +/− SD. *P < 0.05 in Student's *t*‐test. One representative experiment of three is shown. (B) T cells were prepared from TNFR1‐deficient mice and transferred into TNFR1‐deficient mice. Anti‐CD3ϵ‐antibody was given in an i.v. injection. Data from 3–4 mice per group are shown as mean +/− SD. *P < 0.05 in Student's *t*‐test. The experiment was performed once. (C) T cells were prepared from C57BL/6 mice and transferred into TNFR2‐deficient mice. Anti‐CD3ϵ‐antibody was given in an i.p. injection. Data from three mice per group are shown as mean +/− SD. * indicates *P* < 0.05 in Student's *t*‐test. One representative experiment of two is shown.

Adoptively transferred TNF‐deficient T cells into TNF‐deficient septic mice were slightly reduced in their proliferation compared to cells transferred into TNF‐deficient naïve mice, though the effect was not statistically significant. Thus, sepsis‐induced suppression seems to be TNF‐dependent (Fig. 4A). However, T cell proliferation of adoptively transferred cells was clearly reduced in septic mice deficient of either TNFR1 or TNFR2 (Fig. 4B and C). Thus, while TNF plays a role in the reduced T cell proliferative capacity after sepsis, neither the function of TNFR1 nor of TNFR2 alone solely accounts for this effect.

### Treg cells are involved in sepsis‐induced suppression

**Figure 5:**
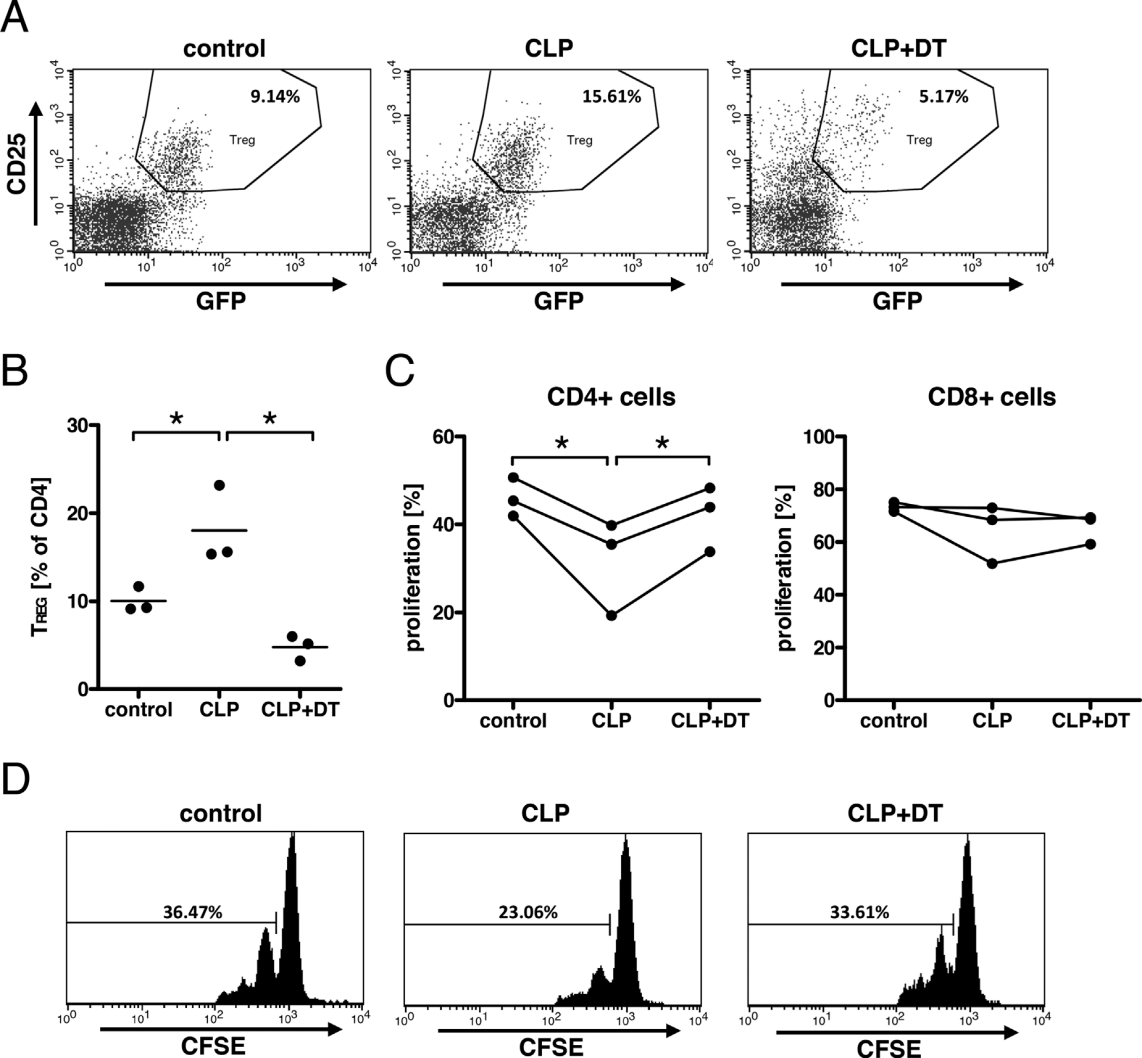
Depletion of Treg cells restored T cell proliferation after CLP. Splenocytes were prepared from untreated control C57BL/6 mice. T cells were separated from the splenocytes, labeled with CFSE, and injected into untreated DTR‐Foxp3‐GFP mice (control), DTR‐Foxp3‐GFP mice 2 days after CLP (CLP), and DTR‐Foxp3‐GFP 2 days after CLP that have been treated with diphtheria toxin (CLP+DT). The next day, mice received an i.v. injection of anti‐CD3ϵ‐antibody. Three days after injection, splenocytes were harvested and analyzed by flow cytometry for regulatory T cells (A and B) and proliferating cells of the CFSE‐positive population (C and D). (A) Density plots of one representative mouse per group. Treg are identified as GFP^+^ (i.e. Foxp3^+^) and CD25^+^ cells. (B) Each symbol represents one mouse, line shows the mean. The data shown in A and B is from a representative experiment of three repeats. (C) Each symbol represents one experiment (mean of 3–4 mice); the respective experiments are connected by lines. * indicates a statistically significant difference in a paired one‐way ANOVA with Tukey's posttest. (D) Histograms of the CFSE‐positive CD4^+^ T cells of one representative mouse per group of a representative experiment. The horizontal line indicates the marker defining proliferating cells.

We investigated the impact of Treg on the reduced T cell proliferation following sepsis as we have previously noted an increased frequency of Treg following sepsis 15. Here, we used a mouse model system that expresses the diphtheria toxin receptor (DTR) under the control of the Foxp3‐promoter (DTR‐Foxp3‐GFP mice). The selective expression of the DTR enables selective depletion of Treg by application of diphtheria toxin. Similar to wild‐type mice, we observed an increased frequency of Treg in the splenic CD4 population of septic DTR‐Foxp3‐GFP mice (Fig. 5A and B). The successful depletion of Treg (Fig. 5A and B) prevented the suppression of T cell proliferation in septic DTR‐Foxp3‐GFP‐recipient mice (Fig. 5C and D), indicating a role for Treg in the suppression of T cell functions in sepsis.

## Discussion

In previous work, we and others demonstrated impaired functions of myeloid cells and B cells following sepsis 14, 16, 19, 20, 21. In addition, T cell dysfunction was demonstrated and accompanied by increased numbers of Treg 22, 23, 24. However, while impaired antigen‐dependent T cell proliferation was Treg independent, 25 it could be overcome by agonistic anti‐GITR antibody treatment 26. In this study, we add to these findings by investigating the impact of sepsis on T cell proliferative responses provided by the septic environment.

An important aspect in our study is the method of T cell activation. We aimed at circumventing the detrimental effects sepsis has on antigen‐presenting cells. An anti‐CD3ϵ‐antibody activates T cells by engaging the CD3ϵ molecule, which starts the TCR signaling pathway. Hence, this treatment follows the natural activation of T cells closely. Importantly, other common antigen‐independent activators (such as Concanavalin‐A and PMA/Ionomycin) act on many cell types, while anti‐CD3ϵ‐antibodies are T‐cell‐specific. Superantigens act by linking the MHC with the TCR, involving antigen‐presenting cells in the T cell activation. However, Fc‐receptors on many cell types affect the availability and function of the anti‐CD3ϵ‐antibody, and costimulatory molecules expressed on antigen presenting cells impact the T cell activation. Therefore, while we aimed at reducing the direct involvement of other cell types on the T cell activation, no method we know of can act completely T cell specifically. Of note, we have previously shown that CD11b^+^ and CD11c^+^ antigen‐presenting cells show a tendency to increased levels of costimulatory markers 16. Hence, the levels of costimulation should not be an aspect of the reduced T cell proliferation we observed in this study.

Our data show a detrimental effect of sepsis on in vivo T cell proliferation. This effect is independent of antigen‐specific activation and can be observed even after antigen‐independent anti‐CD3ϵ activation in vivo. This effect was readily observed in the spleen, but only weakly (and not statistically significant) in the peripheral lymph nodes. As we wanted to assess the systemic effects of sepsis, we did not analyze the peritoneum‐draining lymph nodes. Interestingly, the T cells show an elevated activation state after sepsis, as we have shown previously by the activation marker CD69 16 and now here by the activation marker CD25. The expression of CD25 needs to be carefully interpreted. While CD25 is a valid activation marker for CD8^+^ T cells, both activated CD4^+^ T cells and regulatory T cells express CD25. Here, the increased levels of CD25‐positive CD4^+^ T cells point to an increase in Treg after sepsis, as demonstrated by us previously 15.

The suppression of T cell function is not an intrinsic property of the T cells following sepsis. Instead, the environment in the septic hosts impairs T cell proliferation. This is demonstrated by the restoration of the proliferative function of T cells from septic mice after their transfer into normal non‐septic mice. Additionally, we observed restored proliferative capacity of T cells from septic animals in vitro. It should be mentioned that other studies have shown a reduced proliferative capacity of T cells in vitro following sepsis (e.g. 26). We suggest that the controversial finding is based on the use of CFSE in our study. CFSE as a marker for proliferation allows studying the whole proliferative response while the incorporation of ^3^H‐thymidin only highlights the DNA synthesis during a limited time window. Furthermore, the fact that both, naïve T cells and T cells from septic mice, are equally suppressed in septic mice but not in naïve control mice after adoptive transfer demonstrates that T cells from septic mice are not permanently damaged but experience a suppressive effect exerted by the septic host environment. This is an interesting difference to the suppression of DC function, where sepsis was shown to lead to long‐lasting epigenetic alterations regulating DC function 20.

The immune environment is formed by the inflammatory response accompanying a septic insult with changes such as high serum levels of inflammatory cytokines paralleled by a transiently suppressed cytokine production capacity 11, 12, 13 and induction of suppressive cell types such as MDSC 17 and Treg 13, 27. In our study, TNF‐deficient mice did not show suppressed T cell proliferative capacity following sepsis clearly demonstrating the important role of TNF in sepsis‐induced suppression. However, the suppression was still observable irrespective of whether only TNFR1 or TNFR2 was available for activation by TNF. This demonstrates that it does not make a difference which of the two TNF receptors transduces the TNF effect for T cell suppression following sepsis. Interestingly, TNF and particularly TNFR2 have previously been shown to be involved in the development of Treg after sepsis 15. In this study, depletion of Treg rescued T cell proliferation after sepsis. The induction of Treg is a classical effect in sepsis and Treg cells are potent inhibitors of T cell functions, both directly and indirectly by acting on antigen‐presenting cells. Here, the Treg cells act probably directly on the T cells, as these cells are activated (as seen by expression of CD25), but fail to properly enter proliferation even after antigen‐independent activation. These results are also in line with the finding of Sade‐Feldmann et al. showing that TNF manipulates the immune system toward the generation of a MDSC‐based suppressive environment in cancer 28. This observed immunosuppression was rather due to environmental factors than to intrinsic defects in the immune cells. As the activation of the TNFR2 has been shown to play a critical role for both Treg and for MDSC, 15 participation of MDSC in the sepsis‐induced suppression needs further investigation.

In conclusion, this study demonstrated that the reduced T cell function following sepsis is mediated by activation of the TNF–TNF receptor system and suppression by Treg cells. The T cells regain their functional capacities after removal from the septic environment or after depletion of regulatory T cells. Hence, the immune modulatory events following the septic insult do not alter the T cell population directly. This completes our picture of sepsis, where the septic event leads to a release of pro‐inflammatory as well as anti‐inflammatory cytokines, epigenetic impairment of antigen‐presenting cells and induction of regulatory cells, causing immunosuppression.

## Materials and Methods

### Mice

C57BL/6 mice were purchased from Janvier (Le Genest, France). TNFR1‐deficient mice (C57BL/6‐*Tnfrsf1a^tm1Imx^*/J) 26 and TNFR2‐deficient mice (C57BL/6‐*Tnfrsf1b^tm1Mwm^*) 29 were purchased from The Jackson Laboratory (Bar Harbour, ME, USA), and CD45.1 (C57BL/6J Ly5.1) 30 from Charles River (Sulzfeld, Germany). TNF‐deficient mice (B6.TNF^−/−^) 31 and OT‐II female mice, transgenic for a T cell receptor that specifically recognizes ovalbumin (OVA) residue 323‐339 peptide in the context of the MHC class II I‐Ab molecule 32, were bred in the animal facility of the University of Regensburg. Deficiency of TNF, TNFR1, and TNFR2 expression was verified by PCR. DTR‐Foxp3‐GFP mice (B6.129(Cg)‐*Foxp3^tm3(DTR/GFP) Ayr^*) express the diphtheria toxin receptor and GFP under the Foxp3 promoter 33 and were bred in the animal facility of the University of Regensburg. Mice were housed in the animal facility of the University of Regensburg and handled in accordance with institutional guidelines (Az:54‐2532.1‐27/10).

### CLP

Cecal ligation and puncture has been described previously 13. Mice were anesthetized with 0.75 mg/kg Ketanest (Park, Davis & Company, Munich, Germany) and 16 mg/kg Xylazin (Bayer AG, Leverkusen, Germany) i.p. The caecum was exteriorized and about 30% of the distal end was ligated and punctured once with a 27G needle to achieve a sub lethal CLP.

### Anti‐CD3‐antibody treatment

Mice were treated with 10 μg anti‐CD3‐antibody (clone 145.2C11 purified from hybridoma supernatant), solved in 200 μl DPBS, by i.p. or i.v. injection. Treatment was performed one day after adoptive transfer of the T cells. Analogous effects were observed with both routes of injection. According to the BD Biosciences, this clone can be used for in vivo activation of T cells.

### Immunization

Mice were immunized with 100 μg OVA, plus 50 μl adjuvants (Imject Alum, Pierce Biotechnology, Rockford, IL, USA) in 25 μl DPBS by s. c. injection.

### Adoptive T‐cell transfer

CD4^+^ and CD8^+^ T cells were isolated from splenocytes by magnetic bead separation (Miltenyi Biotec, Bergisch Gladbach, Germany), achieving a purity around 95%, and labeled with CFSE by incubation in a 2 μM CFSE/DPBS FCS 1% solution for 10 min. Between 1.5 × 10^6^ and 10^7^ CFSE‐labeled cells, resuspended in 200 μl DPBS, were injected i.v. in recipient mice. Three days after anti‐CD3 antibody treatment, spleens as well as axial, brachial, and inguinal lymph nodes were harvested from mice and analyzed.

### DT depletion of Foxp3 cells

Treg depletion in DTR‐Foxp3‐GFP mice was achieved by i.p. injection of 0.5 μg diphtheria toxin per 20 g mouse body weight, solved in 200 μl sterile DPBS, 4 h and 2 days after adoptive cell transfer.

### Flow cytometry

In some experiments, single cell suspensions were prepared from spleen and pooled lymph nodes, and red blood cells were lysed. For other experiments, cells were harvested from cell culture. Unspecific antibody binding was blocked by anti‐FcRII/III‐antibody and cells were stained with fluorochrome‐labeled antibodies. For intracellular cytokines, cells were fixed and permeabilized with a Cytofix/Cytoperm kit according to the manufacturer's instructions (BD Biosciences, Heidelberg, Germany). PerCP‐CD4 (clone RM4‐5), AF647‐CD8 (clone 53‐6.7), APC‐CD25 (clone PC61) and PE‐IFNγ (clone XMG1.2) were purchased from BD Biosciences, eBioscience (Frankfurt am Main, Germany) and Biolegend (Fell, Germany).

### Immunohistology

For immunohistology, spleens from control mice or mice exposed to CLP were harvested after adoptive transfer of CD45.1 T cells (not labeled with CFSE), shock frozen, and stored at −80°C. Sections were prepared in a cryostat. After fixation with acetone, cryosections of tissues were equilibrated in PBS with 0.05% Tween 20 (PBS‐Tween) and blocked with PBS containing 10% FCS and 10% normal mouse serum. After washing with PBS‐Tween, sections were stained with Alexa‐Fluor‐647‐CD4 (clone RM4‐5) and −CD8 (clone 53‐6.7), PE‐B220 (clone RA3‐6B2), and biotin‐CD45.1 (clone A20) antibody (all antibodies purchased from BD Biosciences, eBioscience and Biolegend). Following a secondary step with FITC‐streptavidin, sections were mounted with Permafluor. Pictures were analyzed using AxioVision 4.8 software (Zeiss).

### In vitro activation of T cells

Single cell suspensions from spleens were prepared and red blood cells lysed. For proliferation assays, T cells were separated from splenocytes and labelled with 1 μM CFSE (Invitrogen Life Technologies, Darmstadt, Germany). 5 × 10^4^ purified T cells were cultured with 25 × 10^4^ unlabeled total splenocytes at 37°C in 96‐well plates either without stimulation or with varying concentrations of anti‐CD3ϵ antibody (clone 145.2C11 purified from hybridoma supernatant). The antibody was added soluble to the culture to mimic the injection of soluble antibody in vivo. Cell proliferation was analyzed after 72 h by assaying CFSE dilution by flow cytometry. For detection of cytokine‐producing cells, 4 × 10^5^ total splenocytes were cultured at 37°C in 96‐well plates with anti‐CD3ϵ antibody for 24 h with Brefeldin A added for the last 14 h.

## Conflict of Interest

The authors declare no financial or commercial conflict of interest.
